# Quality of surgical scrub in a heart hospital: Do not take it for granted

**DOI:** 10.15171/jcvtr.2017.28

**Published:** 2017-09-30

**Authors:** Leila Abdollahi, Jafar Sadegh Tabrizi, Ahmadreza Jodati, Naser Safaie, Mohammad Moradi-Joo, Amin Daemi

**Affiliations:** ^1^Student Research Committee, Tabriz University of Medical Sciences, Tabriz, Iran; ^2^Health Services Management Research Center, School of Management and Medical Informatics, Tabriz University of Medical Sciences, Tabriz, Iran; ^3^Cardiovascular Research Center, Tabriz University of Medical Sciences, Tabriz, Iran; ^4^Cancer Research Center, Shahid Beheshti University of Medical Sciences, Tehran, Iran; ^5^Department of Health Management & Economics, School of Public Health, Tehran University of Medical Sciences, Tehran, Iran; ^6^Health Management and Economics Research Center, Iran University of Medical Sciences, Tehran, Iran

**Keywords:** Surgical Scrubbing, Clinical Audit, Hand Hygiene, Infection Control

## Abstract

***Introduction:*** The role of scrub in the prevention of post-surgery infections is well-known. This study aimed to investigate the inputs and process of surgical scrub in operating rooms of the largest heart hospital of northwest Iran.

***Methods:*** This study took place with a before-after design as a clinical audit in 2014. A check list developed based on national and international standards of surgical hand scrub was used as the study instrument. Checklists were completed by observation of surgical team scrubbing in real situation. Descriptive statistics and graphs were used to describe the results.

***Results:*** A compliance degree with the standards for prerequisites, equipment, general items, process and time of scrub was observed as 58%, 55%, 33%, 68% and 22%, respectively. The compliance degree after the intervention was 72%, 66%, 66%, 85% and 61%, respectively. Improvement was observed in all studied aspects of scrub. The total score of compliance with the standards changed from 47% to 70%. The main issues were incorrect order of scrubbing the areas of the hands, incorrect way of scrubbing the arms, insufficient scrubbing the arms (not above elbow), and lack of awareness about hospital’s policy on scrub time.

***Conclusion:*** The results showed defects in the surgical scrub of the studied hospital and that the compliance with the standards can be improved by simple interventions. Periodical audit and observation of the scrub and then feedback is recommended.

## Introduction

Despite considerable advancements in surgery, post-surgery infection still is one of the causes of mortality and the surgical team’s hands are one of the most important causes of these infections.^1^ Hands are caring instruments but they also can be carrier of infections.^2^ According to the World Health Organization (WHO), 40% of the infections seen after healthcare services in the developing countries are preventable.^3^

Hand hygiene is the most fundamental principle and one of the first measures in reducing nosocomial infections and increasing patient safety.^3^ The Center for Diseases Control (CDC) reported that correct hand-washing reduces nosocomial infections by 30%.^4^ The Guide of National Nosocomial Infections Surveillance System has emphasized the correct hand washing before surgical operations.^2^

The purpose of hand washing is to remove all microorganisms on the hands and arms.^5^ In the scrub, the mechanical hand washing is used to eliminate contaminations and to deactivate the microbial flora on the hands.^2^ The scrub of hands reduces the microorganisms to a large extent^2^ and is one of the care standards of surgery.^6^ Doing hand scrub before surgery is emphasized for reducing nosocomial infections related to health professionals.^7^ Comparison of three methods of surgical scrub: 2, 4 and 6minutes showed that 4 minute scrub is more suitable.^8^ The WHO has approved 2 to 5 minutes for scrub’s time.^9^

Considering the size of the incision area and the amount of work in open heart surgery, these surgeries had higher risk of infection.^10^ So, special control of infection seems necessary in heart operating rooms. Because the surgery site infection in heart surgery increases the mortality.^11^ Thus this study is conducted in a single-specialty hospital of heart which is the largest heart hospital in northwest of the Iran.

Previous studies in Iran are focused on the effectiveness of the scrub solution and the appropriate scrub time in terms of reducing microbial flora of the hands of surgical teams.^4,12^ But with the best of our knowledge, no study is published how the scrub is performed in real situations of operating rooms in Iran. This study intended to investigate the scrub inputs and process in operating rooms of Shahid Madani heart hospital of Tabriz.

## Materials and Methods

This study used a before-after design as a clinical audit in two areas of inputs and process in spring 2014. The study site was operating rooms of the heart specialty Shahid Madani hospital of Tabriz city, East Azerbaijan province, Iran. The hospital is a tertiary care hospital and the largest heart hospital of northwest of Iran.

The audit was performed according to the‏ Audit Cycle.^13^ In the first stage of study, to extract the standards of surgical scrub, related texts from the Ministry of Health and Medical Education (MOHME) standards were investigated. In the second stage, surgical scrub in the participating hospital compared with the standards. Then the non-compliances with the standards were identified by the audit team. Finally, appropriate intervention measures proposed for improving the situation. The interventions implemented and then the scrub studied again by the same standards (re-audit) to determine the effectiveness of the interventions.

The audit team included a surgeon from the hospital, an operating room technician from the hospital, head nurse of the operating rooms of the hospital, the nurse in charge for hospital’s infection control, and professionals of health services management.

The study instrument was a checklist with Yes- No questions. The checklist developed based on the standards of surgical scrub.^8,9,11,14,15^ The checklist consisted of two parts. The first part consisted of 9 items: demographics (profession, working history - years, gender, and educational degree), working shift, number of scrubs of the individual in this working shift, time of beginning and ending scrub. The second part consisted of 57 questions in 4 dimensions including prerequisites of scrub (16 questions), the process of scrub (29 questions), general items (3 questions) and equipment (9 questions).

The checklists were completed by observing the surgical teams’ scrub in real situation. Convenient sampling method applied for sampling. Totally 70 observations of scrub were recorded. Considering that the total number of the surgery personnel is limited in the hospital and due to the existence of the cluster effect,^16^ we had sufficed to the 35 completed checklists before and after the intervention. To control the possible confounding effects of emergency operations on the process and time of scrub, the observations limited to the elective surgeries only. One point was given to each question. The answer “Yes” got a point and the answer “No” had no points. To describe the results, the descriptive statistics and graphs were used by the Microsoft Excel 2013 software.

## Results

Of the 35 observed scrubs in the primary study 28 were the first scrub of the individual in the work shift and 7 were the second scrub. The average time of scrub was 3 minutes and 30 seconds for the first scrub and 3 minutes and 17 seconds for the second scrub of the individual. In the category of inputs of scrub, the average score of compliance with the standards in dimensions of prerequisites, equipment, and general items of scrub were 58%, 55% and 33% respectively. In the category of process, for the dimensions of scrub process and scrubbing time the average score of compliance with the standards were 68% and 22% respectively.

Demographic characteristics of participants in two phases of the study are listed in Table 1.

**Table 1:** Demographic information of the observed personnel at audit and re-audit of surgical scrub in the Shahid Madani heart hospital, Tabriz, Iran: 2014

**Variable**	**Characteristics**	**Audit**	**Re-Audit**	**P-value**
Profession	Operating room technician	37% (n = 13)	20% (n = 7)	0.146^a^
	Nurse	12% (n = 4)	31% (n = 11)	
	Surgeon	51% (n = 18)	49% (n = 17)	
Job experience	Mean	16.48 years	17.05 years	0.757^b^
	Median	20 years	20 years	
	Minimum	1 year	1 year	
	Maximum	30 years	30 years	
Gender	Female	40% (n = 14)	43% (n = 15)	1.00^a^
	Male	60% (n= 21)	57% (n = 20)	

^a^ *P* value is prepared by the McNemar test. ^b^ *P* value based on paired samples *t* test.

The most important non-compliances with the standards are presented in Box 1.

**Box 1. Most important non-compliances with the scrub standards in the Shahid Madani Heart Hospital, Tabriz, Iran: 2014**

- Lack of awareness about hospital’s policy on scrub time Disregard to scratches on the hands and arms and not reporting it
- No hand washing with soap before the scrub
- Not being sure that the gown and gloves are ready for use after scrub
- Some scrubbers did not use brushes, while the hospital’s policy was to use it for the fingernails
- Incorrect order of scrubbing the areas of hands (not starting the scrub from nails and fingertips)
- Incorrect way of scrubbing the arms (not using spin movements)
- Insufficient scrubbing the arms (not above elbow)
- Not drying hands with the two ends of the towel
- No possibility to set the water temperature
- Shortage of scrub sink (only one sink-with two faucets- for four operating rooms)
- Putting used brushes inside the scrub sink
- Inappropriate height of scrub solution container
- The clock was not in eyesight of the scrubbers

Also there was a non-compliance not related to scrub but related to infection control. So it was worthy to report. Some personnel went outside the operating room while they wear specific shoes of the operating room. To resolve the mentioned issues a meeting held by the audit team. In the meeting it was agreed that some interventions be implemented. The interventions were:

- Hospital’s policy about scrub time to be communicated in the meetings of the operating room personnel
- To provide the scrubbers (mainly the surgeons and the head nurse) with feedback about the non-compliances
- To install the scrub education poster beside the scrub sink
- To provide a basket for putting the used brushes
- To lower the height of scrub solution container to make it easy to use
- To change the place of the clock to front of the scrub sink

The interventions implemented and the re-audit was conducted 2 weeks later. Of the 35 observed scrubs, 28 were first scrub of the individual in the work shift and 7 were the second scrub. The average time of the scrub was 4 minutes for first scrubs and 2 minutes and 25 seconds for second scrubs. Results of the re-audit showed that in the category of scrub inputs, the average score of compliance with the standards in dimensions of prerequisites, equipment and general items of scrub were 72%, 66% and 66% respectively. In the category of process, for the dimensions of scrub process and scrubbing time the average score of compliance with the standards were 85% and 61% respectively. Table 2 compares the scores of the audit and re-audit, separated by scrubbers’ profession.

**Table 2:** Comparison of the audit and re-audit scores of the surgical scrub in the Shahid Madani Heart Hospital, Tabriz, Iran: 2014; separated by professions

**Dimensions**	**Stage of audit**	**Operating room technician (%)**	**Nurse (%)**	**Surgeon (%)**
Scrub prerequisites	Audit	59	56	57
	Re-audit	73	70	71
Scrub process	Audit	66	76	68
	Re-audit	76	82	90
Scrub time (first and second)	Audit	15	50	22
	Re-audit	57	72	52

**Figure 1:**
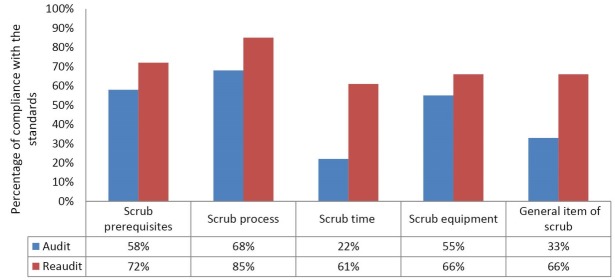
Comparison of total scores of compliance with the standards, before and after the interventions on surgical scrub in the Shahid Madani heart hospital, Tabriz, Iran: 2014.

Figure 1 compares the total scores of compliance with the standards, before and after the interventions.

## Discussion

Results of the primary investigation showed considerable defects in the surgical scrub of the studied hospital. Compliance with the scrub time standards had the lowest score (22%). After implementing interventions, improvement was observed in all studied aspects of scrub. The improvement was bigger in scrub time (39%)

*Scrub prerequisites*: Hospital’s policy on scrub time and on using brushes communicated. This communication resulted in improvements in the time and process areas (Figure 1). Previous studies have shown that increasing awareness about a policy will lead to increased compliance with it.^17^ Lack of awareness about hospital policy leads to variations in the scrub of the personnel.^18^ Providing the surgical staff with the documented policy is recommended^19^. It may help to improve the quality of scrub and then to prevent post-surgery infections.^20^

*Scrub process*: There were several defects in the process of the observed scrubs. A previous study in Iran also stated that the surgical scrub in some cases is performed incorrectly.^4^ The related interventions were to install the educational poster of scrub beside the scrub sink and to feedback the scrubbers. The interventions led to improvements (Figure 1), just like other studies.^17^ Cutter and Jordan stated that the compliance with standard precautions is based mainly on the individual’s perception.^21^ A study in Turkey showed that the scrub performance of the surgical staff is not correlated with their knowledge of infection control. They believed that the proper scrub time (in average) is 4.2 minutes but in practice they scrubbed only for 1.15 minutes. Seventy percent of them said in interview that use of brush is needed for hand disinfection but 73% did not use brushes in practice.^22^ It seems that mandatory formal educational courses do not help and the staff, especially the surgeons, do not attend the courses even when mandatory.^21^ Continuous regular education, providing feedback, and participation of the surgeons in developing the hospital policy may result in elevated compliance to the standards.^21,23,24^

*Scrub time*: According to hospital’s policy, standard time for the first scrub was 5 minutes and for the second scrub was 2 minutes. However, observations of this study showed that the duration of scrub in the first and second scrub of the individuals was almost similar (3 minutes and 30 seconds versus 3 minutes and 17 seconds). In fact when there is no explicit rule about scrub time, everyone’s scrub is based on his/her previous training or experience.^4^ Since over-scrubbing can increase the risk of skin damage,^20^ it is better for the scrubbers to follow the rules. Also the skin damage due to scrub may cause them not to do scrub perfectly.^4,25^ Over-scrubbing also leads to considerable waste of water.^26^ After the interventions implemented, the average time of the scrub (first and second scrub) changed according to the rules (4 minutes for first scrubs and 2 minutes and 25 seconds for second scrubs).

*Scrub equipment*: The scrub solution container was installed very high and as it was automatic, when someone wanted to get solution for scrub, it often shed on the clothes of the scrubber. The container lowered to fix the problem. The observations also made it clear that the scrubbers did not pay attention to scrub time. The clock was behind the scrubbers and thus was visible in mirror. As an intervention to make seeing it easier, the place of clock changed to the front of the scrub sink. This might have an effect in the improvement of scrub time.

*General items of scrub*: The National Institute of Clinical Excellence has suggested that surgeons, surgical assistants and surgical team do scrub on entering to the operating room and do alcohol hand rub between surgeries or after changing clothes.^27^ Yet, in this study the hospital’s policy was to do scrub for every surgery (with different time duration for first and second scrub). This rule was followed by all surgical team members. As an intervention in this dimension, a poster of step-by-step scrub education along with proposed time duration for scrub, attached to the wall beside the scrub sink to remind the scrubbers how and how much to scrub. Next item in this dimension was periodical sampling of scrubbers’ hand for microbial counting. Although the sampling of the hands was mentioned in the national standards, it was not done in the hospital. Only a sampling from the surfaces was done periodically.

Further to the periodical microbial count of the scrubbed hands, the infection control nurse of the hospital can play a more active role in improving the quality of surgical scrub in the hospital. Activities include regular periodical and random observation of the scrubbing, feeding back to the personnel, reporting to the hospital authorities, and presenting the results as statistics and graphs. The infection control staff should be trained on methods of investigating the surgical site infection, and had a basic knowledge of computer and mathematics to be able to provide education and feedback to the personnel.^23^

Post-surgery infection results in lengthened hospital stay, delayed healing, increased use of antibiotics, financial and psychological burden and in some cases to mortality. The most important, most simple and least expensive measure to prevent it, is the hand hygiene of the surgical team.^25^ The present study found considerable defects in the surgical scrub of a teaching hospital of heart specialty. So we should never take the quality of the scrub for granted even in such contexts. On the contrary, due to high sensitivity of open heart surgery, we should pay more attention to it. Low compliance with the scrub standards may be affected by various factors including workload.^28^ We used audit to study and improve the situation as it is reported to able to be a tool for improving the infection control.^29^ Previous studies also recommended observation of surgical scrub behaviors and regular audit and feedback to the surgeons and other surgical team members.^22,23^

## Limitations of the study

This study investigated the inputs and process of the preoperative scrub. It is better to see the outputs too. Studying the output and outcome measures of scrub such as microbial count of the scrubbed hands and the prevalence of post-surgery infection might help us to know if the scrub is effective or not. Another limitation of the study was small sample size. Although the sample size was enough for the study purpose, a larger sample of multi-center study would make the researchers able to perform statistical tests to investigate potential variations in job, time/working shift and any other factor. The observational nature of the study also may cause bias. The scrubbers might have changed their scrub due to presence of the observer.^19^

## Conclusion

This study observed the surgical scrub in the operating rooms of a heart specialty hospital in the real situation. The results showed considerable defects in the scrub of the studied hospital. After implementing some interventions, the compliance with the standards improved in all dimensions. Yet, the gap between compliance scores and the expected situation shows that still there is room for improvement. Considering the results of the study, we recommend periodical review of the scrub in the operating rooms of the hospitals to see whether it complies with the standards or not and to point out the problems to be solved.

## Ethical approval

Study protocol approved by the Institutional Review board which is the Ethics Committee of Tabriz University of Medical Sciences. The committee did not require consent. Permission of the hospital authorities attained.

## Competing interests

All authors declare no competing financial interests exist.

## Acknowledgements

This study is funded by the Students Research Committee of Tabriz University of Medical Sciences. Authors also would like to thank Mr. Mehdi Banagozar, Mr. Mohammad Ali Sheikhalizadeh, and Mrs. Roghayeh Mahmoudian, the personnel of the Shahid Madani hospital of Tabriz for their kind cooperation.
